# Transcriptome Profiling of Dysregulated GPCRs Reveals Overlapping Patterns across Psychiatric Disorders and Age-Disease Interactions

**DOI:** 10.3390/cells10112967

**Published:** 2021-10-31

**Authors:** Roudabeh Vakil Monfared, Wedad Alhassen, Tri Minh Truong, Michael Angelo Maglalang Gonzales, Vincent Vachirakorntong, Siwei Chen, Pierre Baldi, Olivier Civelli, Amal Alachkar

**Affiliations:** 1 Department of Pharmaceutical Sciences, School of Pharmacy, University of California Irvine, Irvine, CA 92697, USA; rvakilmo@uci.edu (R.V.M.); walhasse@uci.edu (W.A.); trimt2@uci.edu (T.M.T.); michaeg5@uci.edu (M.A.M.G.); vvachira@uci.edu (V.V.); ocivelli@uci.edu (O.C.); 2 Department of Computer Science, School of Information and Computer Sciences, University of California Irvine, Irvine, CA 92697, USA; siweic@uci.edu (S.C.); pfbaldi@uci.edu (P.B.); 3 Institute for Genomics and Bioinformatics, School of Information and Computer Sciences, University of California Irvine, Irvine, CA 92697, USA; 4 Center for the Neurobiology of Learning and Memory, University of California Irvine, Irvine, CA 92697, USA

**Keywords:** GPCRs, psychiatric disorders, transcriptomics, age-dependent expression

## Abstract

G-protein-coupled receptors (GPCRs) play an integral role in the neurobiology of psychiatric disorders. Almost all neurotransmitters involved in psychiatric disorders act through GPCRs, and GPCRs are the most common targets of therapeutic drugs currently used in the treatment of psychiatric disorders. However, the roles of GPCRs in the etiology and pathophysiology of psychiatric disorders are not fully understood. Using publically available datasets, we performed a comprehensive analysis of the transcriptomic signatures of G-protein-linked signaling across the major psychiatric disorders: autism spectrum disorder (ASD), schizophrenia (SCZ), bipolar disorder (BP), and major depressive disorder (MDD). We also used the BrainSpan transcriptomic dataset of the developing human brain to examine whether GPCRs that exhibit chronological age-associated expressions have a higher tendency to be dysregulated in psychiatric disorders than age-independent GPCRs. We found that most GPCR genes were differentially expressed in the four disorders and that the GPCR superfamily as a gene cluster was overrepresented in the four disorders. We also identified a greater amplitude of gene expression changes in GPCRs than other gene families in the four psychiatric disorders. Further, dysregulated GPCRs overlapped across the four psychiatric disorders, with SCZ exhibiting the highest overlap with the three other disorders. Finally, the results revealed a greater tendency of age-associated GPCRs to be dysregulated in ASD than random GPCRs. Our results substantiate the central role of GPCR signaling pathways in the etiology and pathophysiology of psychiatric disorders. Furthermore, our study suggests that common GPCRs’ signaling may mediate distinct phenotypic presentations across psychiatric disorders. Consequently, targeting these GPCRs could serve as a common therapeutic strategy to treat specific clinical symptoms across psychiatric disorders.

## 1. Introduction

**Figure 1:**
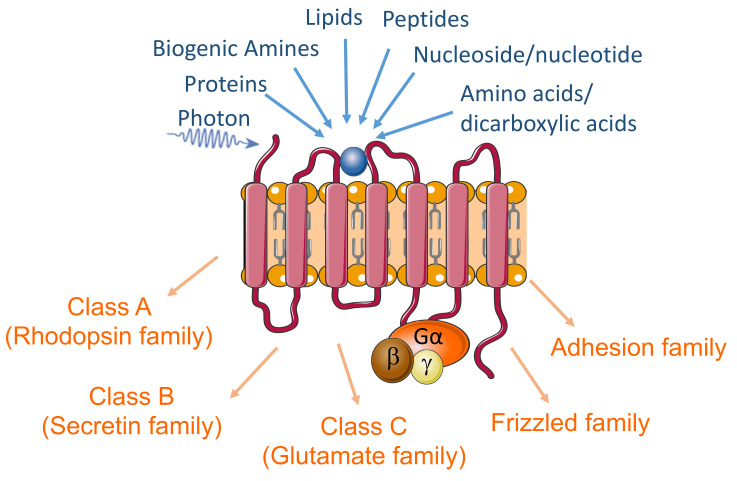
The diagram highlights the GPCRs’ seven transmembrane structure, primary endogenous ligands, and the sub-classes of these receptors.

G protein-coupled receptors (GPCRs) comprise the largest family of cell-surface receptors (>800 in the human genome) and mediate the transduction of extracellular signals into intracellular responses [1]. GPCRs signaling systems, consisting of the heterotrimeric G-protein subunits, GPCRs, and downstream effector molecules, can transduce molecular signaling of a wide variety of endogenous ligands, including hormones, neurotransmitters, proteins, lipids, and peptides (Figure 1). They also mediate cells’ response to exogenous and environmental ligands and stimuli such as photons, tastants, and odorants. Through modulation of cell response to extracellular signaling molecules, GPCRs regulate numerous cellular functions (e.g., metabolism, proliferation, migration) and modulate cells’ interactions and responses to their environment, which implicate this superfamily in almost all physiological functions and pathological processes [2]. Disruptions of GPCRs have been implicated in the pathophysiology of a broad spectrum of diseases ranging from psychiatric disorders to cancer [3,4,5,6,7,8,9,10,11]. Further, the GPCRs constitute the largest family of therapeutic targets, accounting for around 34% of all Food and Drug Administration (FDA)-approved drugs [12,13].

Psychiatric disorders including schizophrenia (SCZ), autism spectrum disorder (ASD), bipolar disorder (BP), and major depressive disorder (MDD) are among the leading causes of disability worldwide, posing enormous burdens on individuals, families, and society [14]. Unfortunately, available pharmacological treatments have poor efficacy, and, currently, there are no drugs that would be considered curative. Furthermore, despite decades of intensive research and considerable success in identifying genetic and environmental risk factors, our limited knowledge of the molecular and cellular mechanisms underlying these psychiatric disorders has hampered effective pharmacological interventions.

A substantial body of evidence supports that abnormalities in the regulation of GPCRs play an integral role in the neurobiology of psychiatric disorders. First, many GPCRs are abundantly expressed in the central nervous system (CNS), particularly in the brain [15,16]. Second, the brain GPCRs constitute the largest receptor superfamily that regulates various neurophysiological responses. GPCRs control brain function by regulating multiple downstream signaling pathways and mediating the actions of all known neurotransmitters [17,18]. Indeed almost all neurotransmitters involved in psychiatric disorders act directly through GPCRs [19,20]. Examples include but are not limited to serotonin and noradrenaline (depression and bipolar disorder) and dopamine (schizophrenia) (for review, see [21,22,23,24,25]). Lastly, GPCRs are the most common targets for drugs currently used to treat psychiatric disorders, and most neuropharmacological drugs are known to regulate GPCR activity in the CNS [21,22]. Although most of these GPCR-targeted medications have been in use to treat psychiatric disorders since the 1950s (For review [26]), our understanding of their mechanisms in modulating the pathophysiology and improving the clinical symptoms remains limited and equally limited is our understanding of the roles of GPCRs in the etiology of these diseases. Therefore, the goal of this study is to perform a comprehensive analysis of the brain transcriptomic signatures of G-protein-linked signaling across the major psychiatric disorders ASD, SCZ, BP, and MDD.

## 2. Methods

### 2.1. GPCR Genes

Genes experimentally probed and identified to be GPCRs were extracted from the database IUPHAR/BPS Guide to PHARMACOLOGY (International Union of Basic and Clinical Pharmacology (IUPHAR)/British Pharmacological Society (BPS) Guide to PHARMACOLOGY) [27]. The IUPHAR/BPS database contains about 800 putative GPCR genes that have been identified in humans, including ~450 sensory GPCRs mediating olfaction, taste, light perception, and pheromone signaling [28]. Our initial list included the non-sensory GPCRs (~350) that mediate signaling by ligands corresponding to small molecules, peptides, and large proteins [29]. We used the classification scheme “GRAFS” [30,31], which divides vertebrate GPCRs into five classes (A, B, C, Frizzled, adhesion, and others). Class A (Rhodopsin family) includes receptors for many small molecules, neurotransmitters, peptides, and hormones. Class B (Secretin family) consists of 15 genes in humans, encoding receptors for ligands/hormones with polypeptide structures of 27–141 amino acid residues. Nine mammalian receptors respond to ligands that are structurally related to one another (glucagon, glucagon-like peptides, glucose-dependent insulinotropic polypeptide, secretin, vasoactive intestinal peptide, pituitary adenylate cyclase-activating polypeptide, and growth-hormone-releasing hormone) [32,33]. Class C (Glutamate family) includes metabotropic glutamate receptors, a calcium-sensing receptor, and GABAB receptors, as well as three taste type 1 receptors) [28]. The frizzled family consists of 10 Frizzled proteins (FZD(1–10)) and Smoothened (SMO). The FZDs are activated by secreted lipoglycoproteins of the WNT family, whereas SMO is indirectly activated by the Hedgehog (HH) protein family, acting on the transmembrane protein Patched (PTCH)). The Adhesion class is phylogenetically related to the B class but differs by possessing large extracellular *N*-termini that are autoproteolytically cleaved from their 7TM domains at a conserved “GPCR proteolysis site” (GPS), [34]). In total, our list of GPCR genes contained 376 genes (290 Class A, 15 Class B, 22 Class C, 11 Frizzled, 33 Adhesion, and 5 Others (Table 1 and Table S1).

### 2.2. Transcriptomic Analysis of GPCR Genes in SCZ, ASD, BP, and MDD

The RNA-Seq data of our initial list of 376 GPCR genes were extracted from the largest publically available transcriptomic datasets for SCZ, ASD, BP, and MDD [35,36]. From the initial list, we obtained the gene expression data for 255 GPCR genes in prefrontal cortex tissue samples (559 SCZ, 51 ASD, 222 BP, and 936 controls) from the study of Gandal et al. [35], which retrieved RNA-seq data from the PsychEncode (PEC, Brain-GVEX study) and CommonMind (CMC) datasets [35,37,38,39]. In addition, gene expression data for 306 genes were retrieved from the transcriptomic dataset of cortical brain tissue (87 MDD and 293 controls), obtained from the study of Gandal et al. [36]. The MDD dataset was created using meta-analyses of microarray data obtained from the Gene Expression Omnibus (GEO), ArrayExpress, or from the study directly conducted by these authors [36,40].

GPCR differentially expressed genes (DEs) in SCZ, ASD, BP, and MDD (FDR < 0.05 and *P* < 0.05) were identified and underwent Fisher’s exact test to examine the enrichment of differentially expressed GPCR genes in SCZ, ASD, BP, and MDD.

Since different G-Protein subunits have been shown to be associated with various psychiatric disorders and their pharmacological therapeutics [41,42,43,44,45], we identified the isoforms of the three G-protein subunits (α, β, γ) that are dysregulated in the four disorders. The original list, obtained from the IUPHAR/BPS database, included 33 genes encoding the isoforms of G-αs, G-αi, G-αq, G-12/13, G-β, and G-γ. Of these 33 genes in the original list, we found differential gene expression data for 27 genes in the ASD, SCZ, and BP transcriptomic dataset and 31 genes in the MDD transcriptomic dataset. The overlap of DEs across the psychiatric disorders was assessed using Fisher’s exact test (at *P* < 0.05).

### 2.3. GPCR Differential Expression with Chronological Age 

RNA-Seq data of GPCR genes dorsolateral prefrontal cortex (DLPC) were obtained from the BrainSpan Atlas of developing human brain web portal under the developmental transcriptome dataset (https://www.brainspan.org/static/download.html, accessed on 10 April 2021) [46]. The dataset included brain samples from 42 healthy individuals, including 19 females and 23 males ranging from 4 months to 40 years old, to cover early infancy, early childhood, late childhood, adolescence, and adulthood stages. We then used linear regression to model the gene expression level as a function of age. Age was treated as a continuous variable expressed in years, ranging from 0.33 (4 months) to 40. Finally, we selected the genes linearly associated with chronological age (*p*-value < 0.05) for further investigation.

### 2.4. Analysis of Age-Disease Interaction 

The lists containing genes from both the transcriptomic and the lifespan datasets comprised 248 genes for ASD, SCZ, and BP and 299 for MDD. We analyzed whether GPCR genes exhibiting differential expression with chronological age are more likely to be dysregulated in any one of the four psychiatric disorders. We used Fisher’s exact test to test whether there was an increased proportion of age-associated GPCR genes in the disease-associated ones compared to randomly selected GPCRs.

## 3. Results

### 3.1. GPCRs List

Using the IUPHAR/BPS database, we identified and verified 376 candidate GPCR genes, which belonged to five classes: 290 class A, 15 class B, 22 class C, 11 frizzled, 33 adhesion, and five others.

### 3.2. GPCRs’ Gene Family Is Overrepresented in Psychiatric Disorders’ Transcriptomes

**Figure 2:**
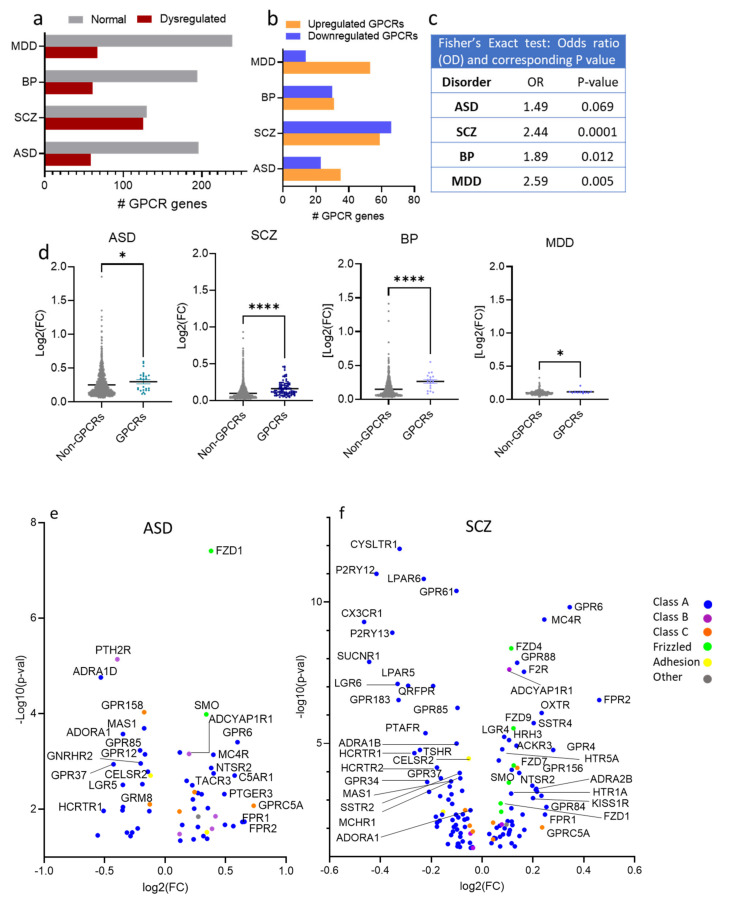

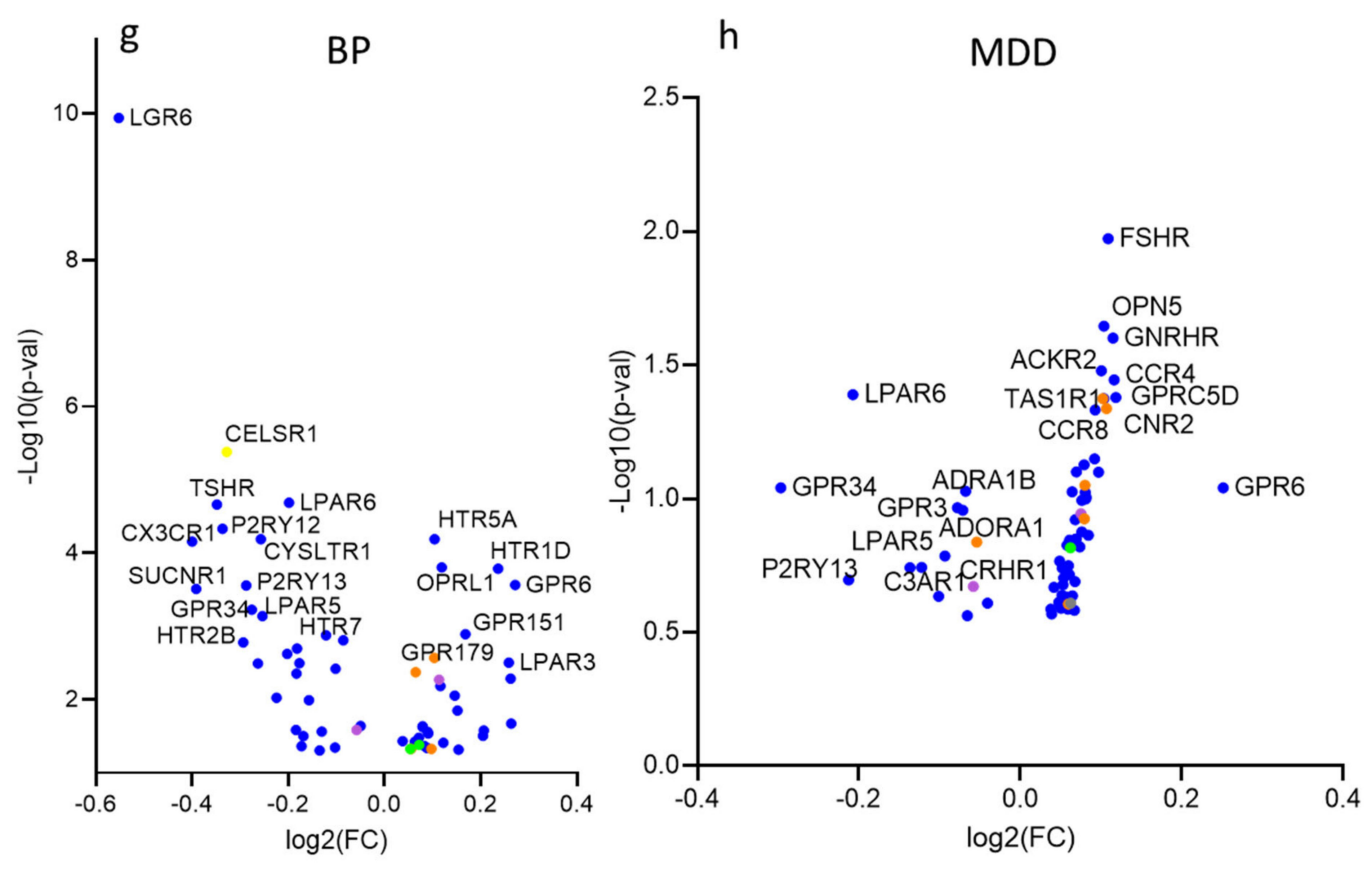
Differential expression of GPCR genes in SCZ, ASD, BP, and MDD; (**a**) Histogram of the distribution of differentially expressed (DE) and non-DE GPCR genes frequencies in ASD, SCZ, BP, and MDD. (**b**) Histogram of the frequency distributions of upregulated and downregulated GPCR genes in the ASD, SCZ, BP, and MDD. (**c**) Fisher’s exact test for the overrepresentation of GPCR genes in the transcriptomes of ASD, SCZ, BP, and MDD. (**d**) Scatter dot plot representing the magnitudes of differential expressions determined by average absolute fold-change of GPCRs and non-GPCRs in ASD, SCZ, BP, and MDD, Mann–Whitney U, *P* = 0.032, <0.0001, <0.0001, =0.03 in ASD, SCZ, BP, and MDD, respectively * *P* < 0.05, **** *P* < 0.0001. (**e**–**h**) Volcano plots of cilia DEGs in (**e**) ASD, (**f**) SCZ, (**g**) BP, and (**h**) MDD.

Of 255 GPCR genes, 190 GPCR genes exhibited dysregulation in at least one of the four disorders: 58, 125, 61, and 67 genes in ASD, SCZ, BP, and MDD, respectively (Figure 2a).

Out of the 58 GPCR genes differentially expressed in ASD, 23 were downregulated and 35 upregulated (Figure 2b). The exact Fisher test revealed an overrepresentation of the GPCR genes in ASD, albeit not significant (OR = 1.49, *P* = 0.069, Figure 2c). The median of the absolute log2(fold change) of ASD GPCR DEs (0.2820) was significantly higher than that of non-GPCR DEs (0.1998) (Mann–Whitney U, *P* = 0.032, Figure 2d). The top upregulated GPCR genes (log2(FC) > 0.2) in ASD were GPRC5A, FPR2, FPR1, GPR6, C5AR1, GPR84, CCR6, PTGER3, GLP2R, TACR3, MC4R, GHRHR, NTSR2, FZD1, EDNRB, APLNR, CELSR1, SMO, OXTR, S1PR1, GPR143, NTSR1, ACKR3, GPRC5C, F2R, ADORA2B, MC1R, and GPR68 (Figure 2e). The top downregulated GPCR genes (log2(FC) < −0.2) in ASD were CHRM5, ADRA1D, HCRTR1, GPR37, PTH2R, HTR1D, LGR5, MAS1, HCRTR2, GPR55, PTGDR2, NMBR, GPR62, GPR149, GPR85, and GNRHR2 (Figure 2e)

Of the 125 GPCR genes differentially expressed in SCZ, 66 were downregulated and 59 upregulated (Figure 2b), and 96 genes passed FDR ≤ 0.05. Exact Fisher analysis revealed significantly overrepresentation of GPCRs in SCZ (Odd ratio OR = 2.44, *P* < 0.0001, Figure 2c). The median of absolute log2(FC) of SCZ GPCR DEs (0.1276) was significantly higher than that of non-GPCR DEs (0.07290) (Mann–Whitney U, *P* < 0.0001, Figure 2d). The top upregulated GPCR genes in SCZ (log2(FC) > 0.2) were FPR2, GPR6, GPR4, GPR84, FPR1, MC4R, GPRC5A, OXTR, NMUR2, LPAR3, P2RY2, ADRA2B, and SSTR4. The top downregulated GPCR genes (log2(FC) < −0.2) were CX3CR1 (Gi/Go), SUCNR1 (Gi/Go, Gq/G11), P2RY12 (Gi/Go), P2RY13 (Gi/Go), LGR6, GPR183, CYSLTR1, LPAR5, HCRTR1, TSHR, LPAR6, PTAFR, GPR34, and RHO (Figure 2f).

In BP, 30 GPCR genes were downregulated and 31 genes upregulated (Figure 2b). The exact Fisher test revealed significant overrepresentation of the GPCR genes in BP (OR = 1.89, *P* = 0.012) (Figure 2c). The median of absolute log2(FC) of BP GPCR DEs (0.27) was significantly higher than that of non-GPCR DEs (0.1069) (Mann–Whitney U, *P* < 0.0001, Figure 2d). The top upregulated GPCR genes in BP (log2(FC) > 0.2) were PTGER1, OPN4, HTR1D, LPAR3, FSHR, FPR1, and GPR6. The top downregulated GPCR genes (log2(FC) < −0.2) in BP are LGR6, CX3CR1, SUCNR1, TSHR, P2RY12, CELSR1, HTR2B, P2RY13, GPR34, GPR183, CYSLTR1, LPAR5, HCRTR1, and PTAFR (Figure 2g).

Out of the 306 GPCR genes, 67 genes (14 genes downregulated and 43 upregulated) were differentially expressed in MDD (Figure 2b), and GPCR genes were also overrepresented in MDD (OR = 2.59, *P* = 0.005, Figure 2c). The median of the absolute log2(FC) of MDD GPCR DEs (0.107) was significantly higher that of non-GPCR DEs (0.097) (Mann–Whitney U, *P* = 0.03, Figure 2d). Only one GPCR gene (GPR6) exhibited log2FC > 0.2, and three genes exhibited log2(FC) < −0.2, GPR34, P2RY13, and LPAR6 (Figure 2h).

### 3.3. Dysregulated GPCRs in Psychiatric Disorders Belong to Specific Receptor Subfamilies

**Figure 3:**
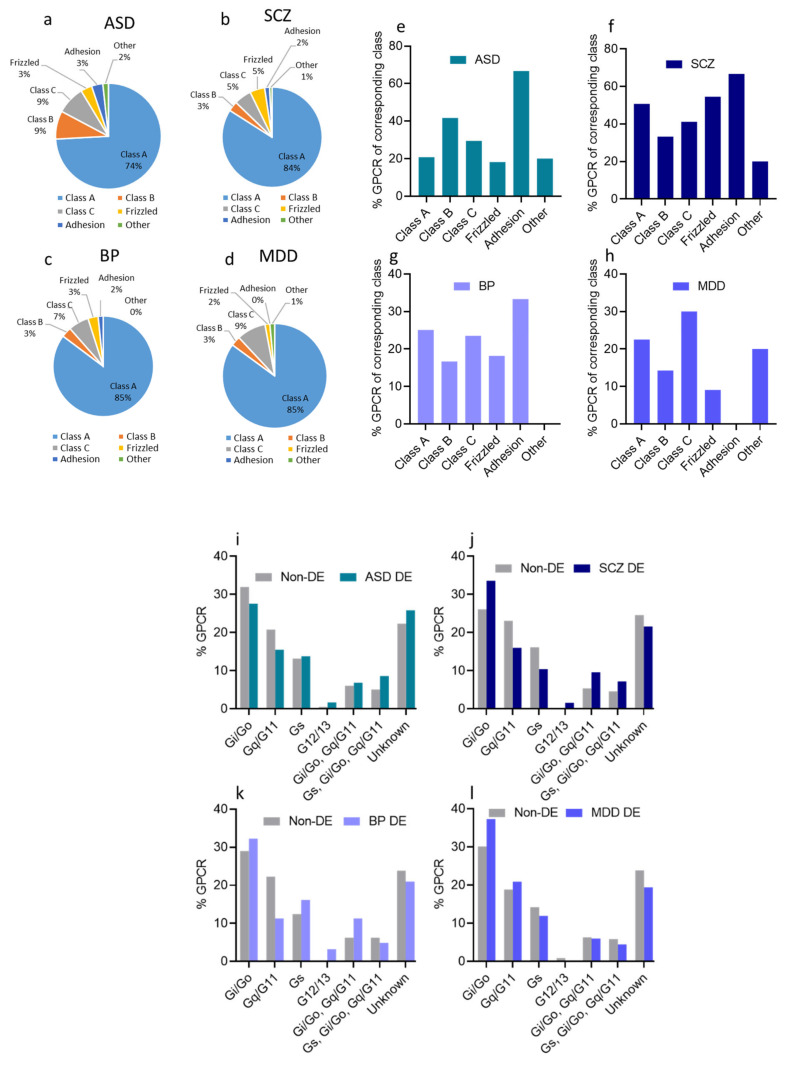

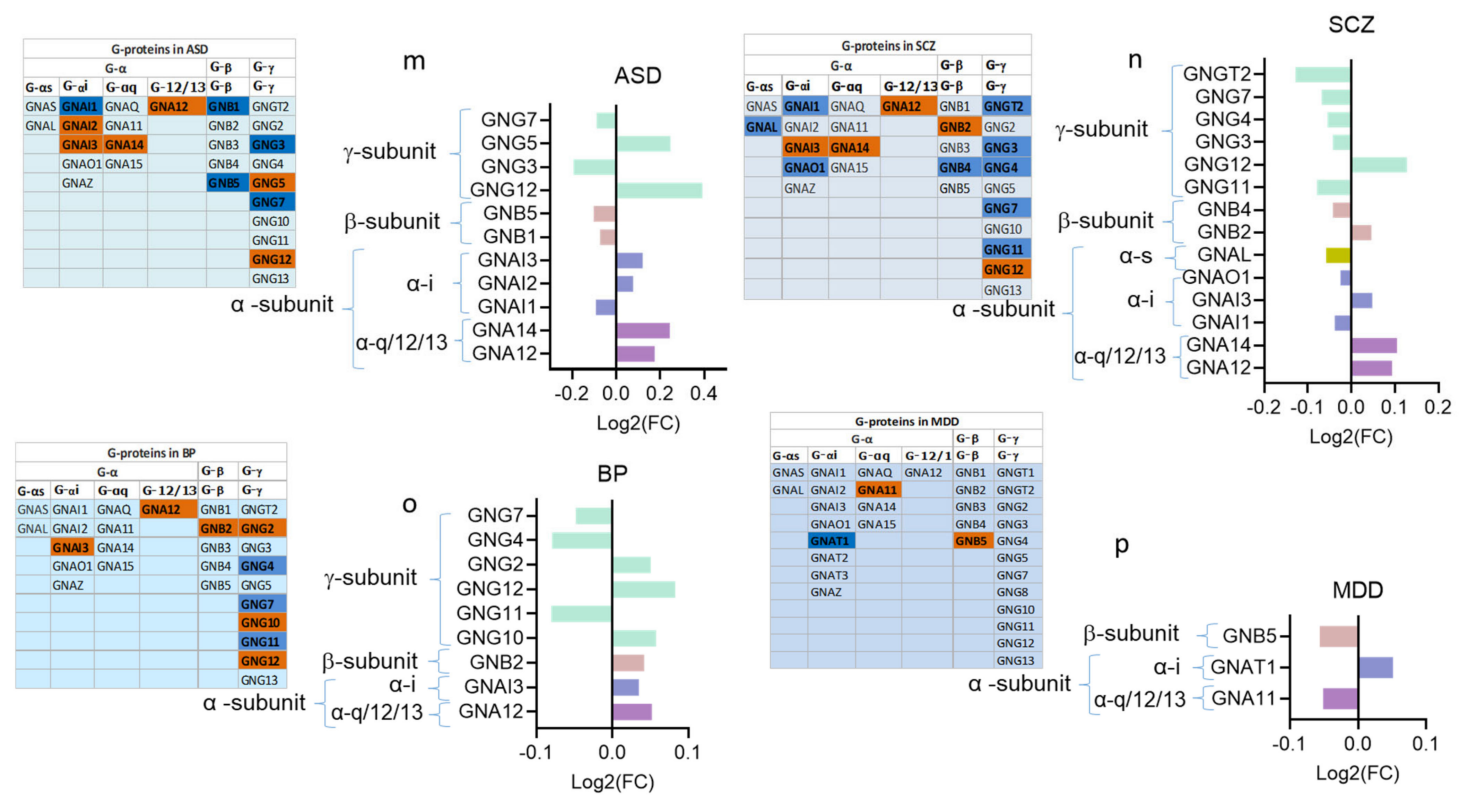
Dysregulated GPCRs in psychiatric disorders belong to specific receptor subfamilies and couple to specific G-proteins; (**a**–**d**) Pie chart showing the distribution of DEs belonging to different GPCR sub-families as proportions of their corresponding total GPCR DEs in (**a**) ASD, (**b**) SCZ, (**c**) BP, and (**d**) MDD. (**e**–**h**) Histogram of the distribution of DEs belonging to different GPCR sub-classes in (**e**) ASD, (**f**) SCZ (**g**) BP, and (**h**) MDD, as proportions of the total subfamily genes. (**i**–**l**). Histogram of the distribution of differentially expressed (DE) and non-DE GPCR genes frequencies to the different G-proteins in (**i**) ASD, (**j**) SCZ, (**k**) BP, and (**l**) MDD. (**m**–**p**) G-protein isoforms’ dysregulations in (**m**) ASD, (**n**) SCZ, (**o**) BP, and (**p**) MDD, (left panel): chart showing different G-protein isoforms of the three G-protein subunits, downregulated: blue, upregulated: red; (right panel): log2(FC) of differentially expressed G-protein isoforms.

As expected, the majority of GPCR DEs in ASD belonged to class A (43 DEs), followed by classes B and C (5 DEs each), and frizzled and adhesion classes (2 DEs each) (Figure 3a). However, when estimated as a percentage of GPCRs within the same GPCR class, adhesion GPCRs exhibited the highest percentage (67% of adhesion GPCRs were DEs in ASD, whereas 21% of class A, 42% of class B, 29% of class C, and 18% of frizzled receptors were DEs in ASD) (Figure 3e).

Similar to GPCR DEs in ASD, the majority of SCZ GPCR DEs belonged to class A (105 DEs), followed by class C (7 DEs), frizzled (6 DEs), class B (4 DEs), and lastly, adhesion GPCRs (2 DEs) (Figure 3b). However, when considering the percentage of DEs within one GPCRs class, adhesion GPCRs seemed to exhibit the highest percentage (67% of adhesion GPCRs were DEs in SCZ, compared to 51% of class A, 33% of class B, 41% of class C, and 55% of frizzled receptors) (Figure 3f).

In BP, 52 DEs belonged to class A, two DEs from class B, four from Class C, two from class frizzled, and one from adhesion GPCRs (Figure 3c). Within the same class analysis, 25% of class A, 17% of class B, 24% of class C, 18% of frizzled receptors, and 33% of adhesion GPCRs exhibited differential expression in BP (Figure 3g).

Lastly, in MDD, we found that 57 DEs were from class A, two DEs from class B, six from class C, one from class frizzled GPCRs (Figure 3d). Within the same class analysis, 23%, 14%, 30%, and 9% of class A, class B, class C, and frizzled GPCRs, respectively, were DEs, and none of the adhesion GPCRs were dysregulated (Figure 3h).

### 3.4. Dysregulated GPCRs in the Four Psychiatric Disorders Couple to Specific G-Proteins

GPCRs are coupled to one of the major types of G-proteins, which are defined according to their alpha subtype class into Gs, Gi/o, Gq/11, and G12/13 (Table S1 [27,47]).

We analyzed the proportion of GPCR DEs based on the G protein, to which they are expected to couple. We found that Gi/o-coupled GPCRs accounted for the most significant proportion of GPCR DEs and non-DEs in the four disorders (Figure 3i–l). G12/13 and Gs-coupled receptors, on the other hand, accounted for a minor proportion among GPCR DEs and non-DEs in the four disorders. Though the proportion of receptors coupling to different G-protein subtypes varied slightly across the four disorders, there was no difference between DEs and non-DEs concerning their coupling to the major G-protein signaling pathways in ASD, SCZ, and MDD. In BP, however, we observed a significantly lower proportion of GPCR DEs that coupled to Gq/11 than the GPCR non-DEs (OR: 10.02, *P* < 0.0001, Figure 3k).

We also identified the isoforms of the three G-protein subunits that were dysregulated in the four disorders. We extracted 27 G-protein isoform genes (12 encoding Gα isoforms, five encoding Gβ isoforms, and 10 encoding G-γ) from the ASD, SCZ, and BP transcriptomic datasets (Figure 3m–p). Of these 27 G-proteins, 11 were DEs in ASD (six upregulated and five downregulated) (Figure 3m), 14 were DEs in SCZ (five upregulated and nine downregulated) (Figure 3n), and nine were DEs in BP (three upregulated and six downregulated) (Figure 3o). The genes encoding G-γ and the G-αi/o isoforms exhibited the highest DEs in the ASD, SCZ, and BP. The MDD transcriptomic dataset contained 32 genes encoding G-protein isoforms, of which only three genes showed differential expression (Figure 3o).

Four genes encoding specific dysregulated G-protein subunits G-α (GNAI3, and GNA12) and G-γ (GNG7 and GNG12) were shared across ASD, SCZ, and BP, and only GNB5 overlapped between MDD and SCZ (Figure 3m–p).

### 3.5. Dysregulated GPCRs Mediate Signal Transductions of Specific Neurotransmitter and Neuropeptide Systems

GPCRs respond to and transduce the signaling of various endogenous and exogenous ligands and stimuli. Endogenous ligands that bind to and activate GPCRs are classified into biogenic amines, cations (including amino acids), lipids, peptides, and glycoproteins. To determine whether the dysregulated GPCRs are more likely to mediate specific signal transduction pathways, we grouped the GPCR DEs in the four psychiatric disorders according to their ligands classes. We found that dysregulated GPCRs coupled to almost all signaling pathways.

**Figure 4:**
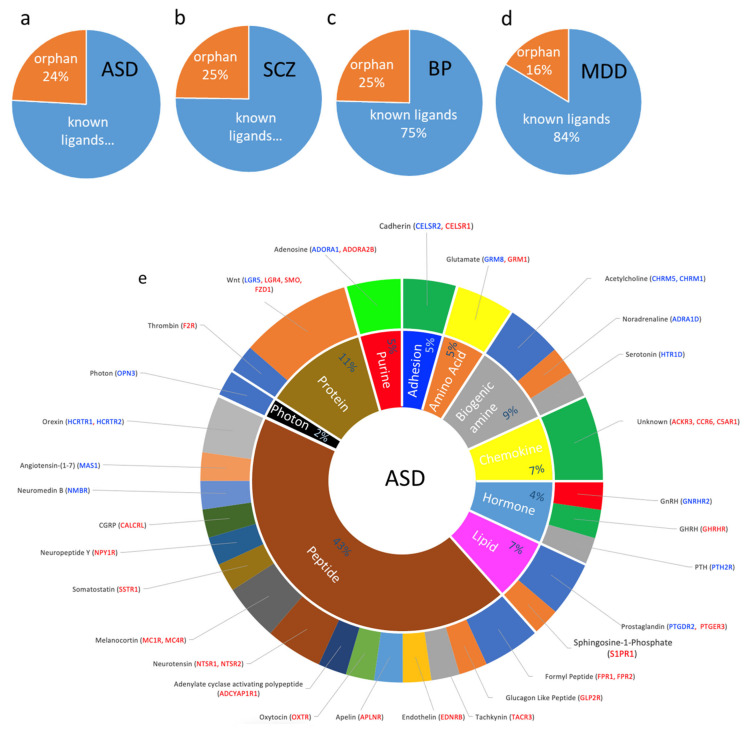

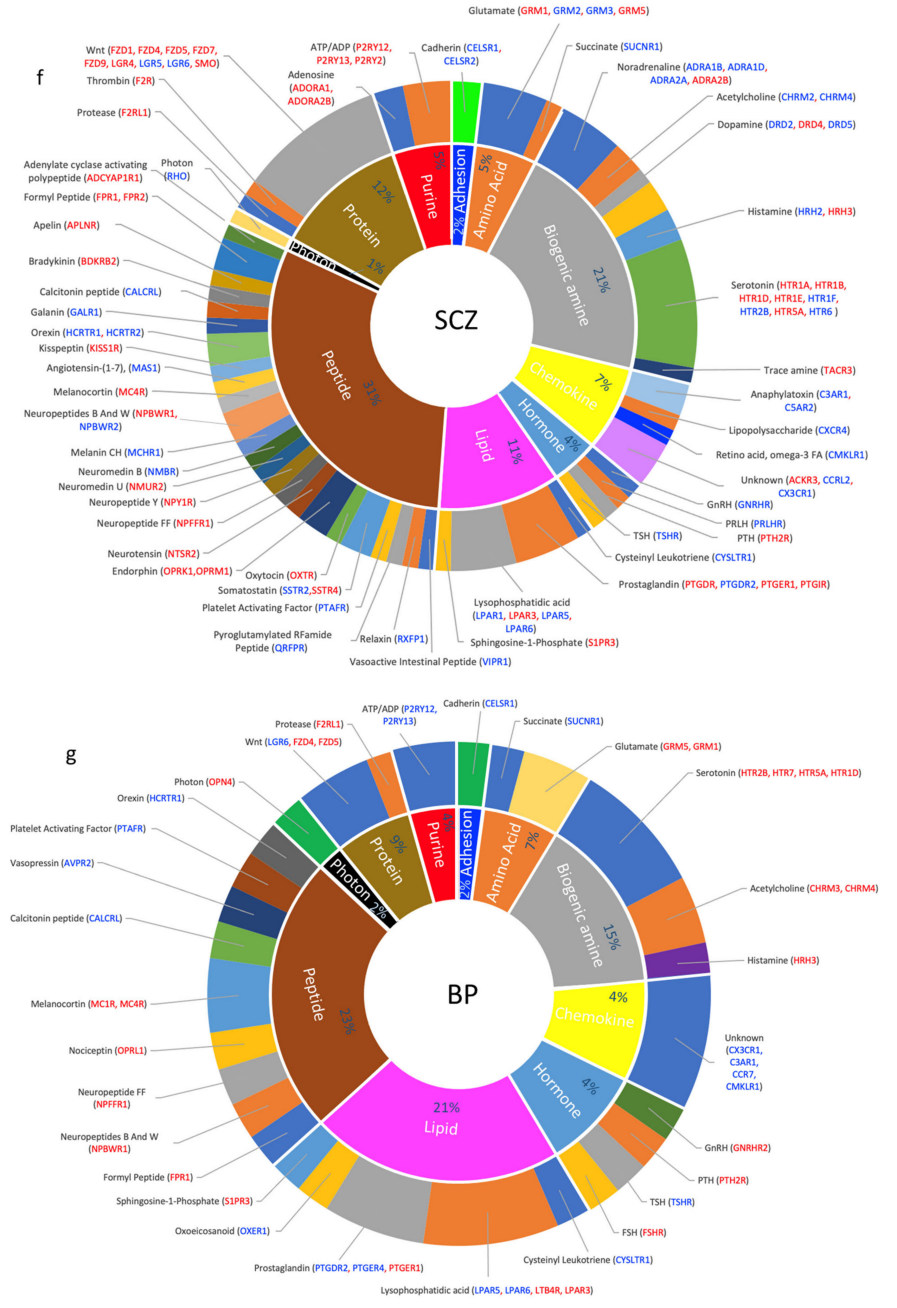

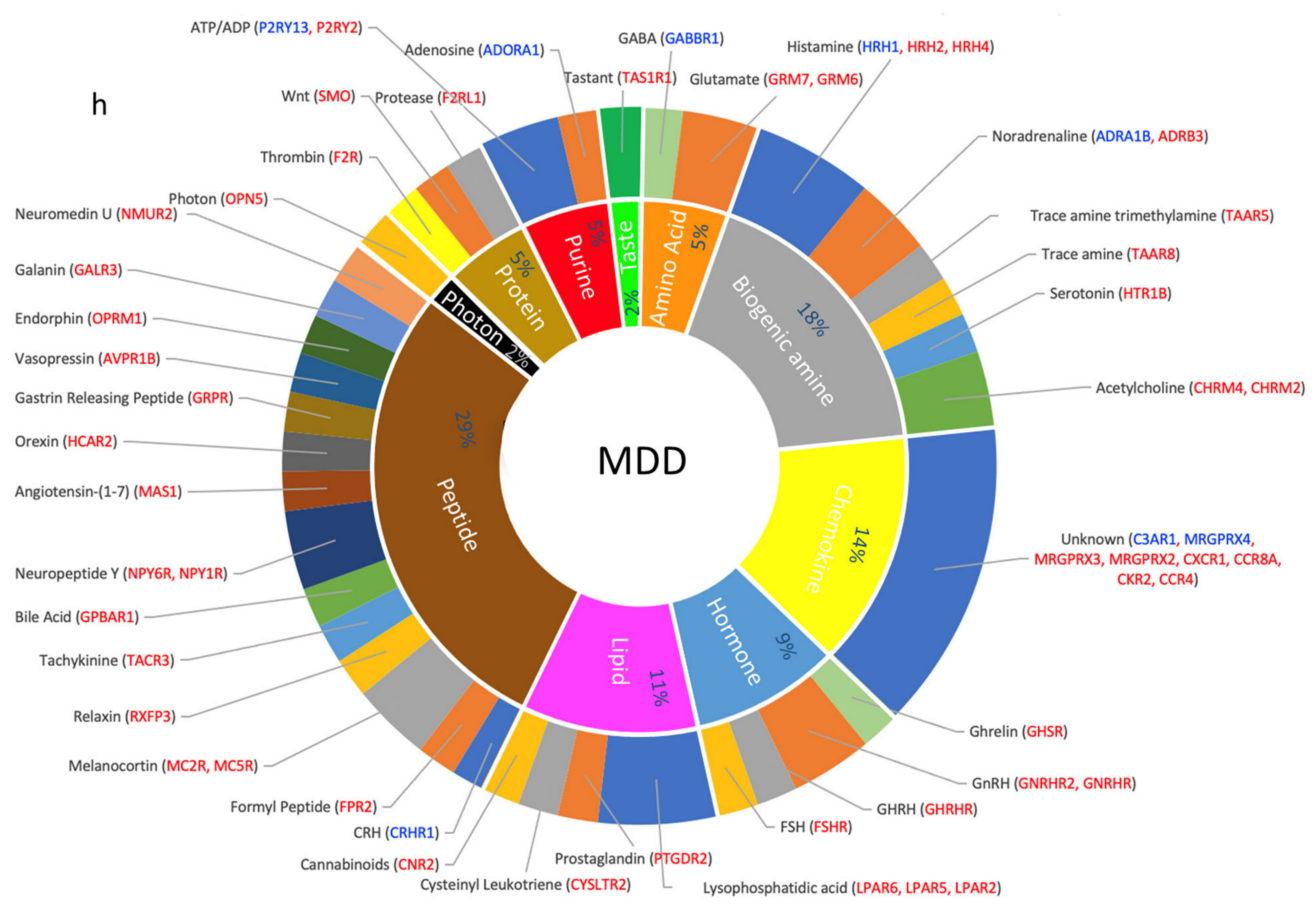
Dysregulated GPCR classifications based on their ligands; (**a**–**d**) Pie chart showing the percentage of DEs belonging to an orphan or non-orphan GPCR as proportions of their corresponding total GPCR DEs in (**a**) ASD, (**b**) SCZ, (**c**) BP, and (**d**) MDD. (**e**–**h**) Doughnut chart showing the percentage of major classes of ligated GPCR DEs in the inner ring and the ligands that activate the GPCR DEs in the outer ring. Differentially expressed GPCRs are shown in blue: downregulated and red: upregulated.

In ASD, orphan receptors comprised 24% of the DEs. The largest number of dysregulated GPCRs belonged to the peptide receptors family (19 DEs) followed by protein (5 DEs), biogenic amine family (4 DEs), hormone, lipid, and chemokine (3 DEs each), and amino acid and purine (2 DEs each) (Figure 4a,e). These include, among others, receptors for orexin, melanocyte-stimulating hormone or melanocortin, neuromedin, neuropeptide-Y, somatostatin, adenylate cyclase-activating polypeptide, neurotensin, oxytocin, apelin, tachykinin, glucagon-like peptide, formyl peptide, endothelin, calcitonin gene-related peptide, and angiotensin 1–7. The protein receptors family included those mediating Wnt and protease-activated receptors (Figure 4e).

Biogenic amines’ receptors dysregulated in ASD included those for the classical neurotransmitters noradrenaline, serotonin, and acetylcholine. In addition, two adenosine receptors (purine family) were dysregulated in ASD. Amino acid receptor DEs included glutamate metabotropic receptors. Endocrine peptide GPCRs dysregulated in ASD included receptors of gonadotropin-releasing hormone, growth hormone-releasing hormone, and parathyroid hormone, and the lipid class included receptors for prostaglandin derivatives and sphingosine-1-Phosphate. Further, 14 dysregulated GPCRs belonged to orphan receptors. Finally, OPN3 was the only dysregulated receptor in ASD (downregulated) that responded to an exogenous ligand (photon).

Similar to ASD, orphan receptors accounted for 25% of the DEs (31 DEs, Figure 4b). The largest number of non-orphaned dysregulated GPCRs in SCZ belonged to the peptide family (30 DEs), followed by biogenic amine (19 DES), protein (11 DEs), lipid (10 DEs), chemokines (7 DEs), and purine (5 DEs), and hormone (4 GPCRs each) (Figure 4f).

Many dysregulated peptide GPCRs found in ASD were also dysregulated in SDZ, including ADCYAP1R1, APLNR, FPR1, FPR2, EDNRB, HCRTR1, HCRTR2, MAS1, MC4R, NMBR, CALCRL, NPY1R, NTSR2, OXTR, and TACR3. Other dysregulated peptide GPCRs in SCZ included receptors of bradykinin, galanin, kisspeptin, melanin-concentrating hormone, neuromedin, neuropeptides B And W, neuropeptide FF, opioids, platelet-activating factor, pyroglutamylated RFamide peptide, relaxin, somatostatin, and vasoactive intestinal peptide. The dysregulated GPCRs that mediate the biogenic amines’ family’s signaling included receptors of all monoamine neurotransmitters: histamine, noradrenaline, dopamine, and serotonin. This family also included acetylcholine receptors. Dysregulated cation receptors included glutamate and succinate receptors (Figure 4f). Lipid receptors included those activated by lysophosphatidic acid, prostaglandins, and sphingosine-1-Phosphate, whereas hormone receptors included those of thyroid-stimulating hormone, gonadotropin-releasing hormone, parathyroid hormone, prolactin-releasing hormone. Purine receptors included receptors for adenosine and ATP/ADP. Proteins receptors were overrepresented in SCZ, including those mediating Wnt signaling, as well as protease-activated receptors. Finally, the photon receptor RHO was downregulated in SCZ (Figure 4f).

Apart from the orphan receptors, which comprised 25% of the DEs (Figure 4c), the dysregulated GPCRs in BP were enriched in the receptors mediating the signaling of peptides (11 DEs), lipids (10 DEs), biogenic amines (7 DEs), chemokines, hormones, and proteins (4 DEs each), cations (4 DEs), and purines (2 DEs) (Figure 4g).

The peptide GPCRs included the opioid peptide OFQ, vasopressin, formyl peptide, orexin, melanocortin, neuropeptides B And W, Neuropeptide FF, CGRP, and Platelet Activating Factor. The lipid family included receptors of prostaglandins, lysophosphatidic acid, oxoeicosanoid, and sphingosine-1-Phosphate. Biogenic amine receptors included receptors that mediate the signaling of acetylcholine, histamine, and serotonin. Hormonal GPCRs included follicle-stimulating hormone, gonadotropin Releasing Hormone, parathyroid hormone, and thyroid-stimulating hormone. Protein dysregulated GPCRs included those of the Wnt pathways. Purine GPCRs included ATP and ADP receptors. As with ASD, and SCZ, one photon receptor (OPN4) was upregulated in BP (Figure 4g).

In MDD, the orphan GPCRs composed only 16% of the DEs (Figure 4d). Non-orphan dysregulated GPCRs included those coupled to the signal transduction of peptides (16 DEs), biogenic amines (10 DEs), chemokines (8 DEs) receptors, hormones (5 DEs), lipids (6 DEs), purines, proteins, and amino acids (3 DEs each) (Figure 4h).

Except for the receptors of three signaling peptides: corticotropin-releasing hormone (CRHR1), gastrin-releasing peptide (GRPR), and bile acid (GPBAR1), all other dysregulated peptide GPCRs in MDD were shared with SCZ: FPR2, GALR3, HCAR2, MAS1, MC2R, MC5R, NMUR2, NPY1R and NPY6R, OPRM1, relaxin RXFP3, and TACR3. Biogenic amine GPCRs included receptors of noradrenaline, acetylcholine, histamine, serotonin, and trace amines. Hormone receptors included follicle-stimulating hormone, gonadotropin-releasing hormone, growth hormone-releasing hormone, and ghrelin. Lipid GPCRs included receptors for cannabinoids, prostaglandin, and lysophosphatidic acid. Purine receptors included adenosine and ATP/ADP receptors. Amino acid GPCRs included glutamate and GABA. In addition, one photon receptor (OPN5) and a taste receptor (TAS1R1) were DEs in MDD (Figure 4h).

### 3.6. Overlap across Psychiatric Disorders of GPCR DEs and Their Signaling Systems

**Figure 5:**
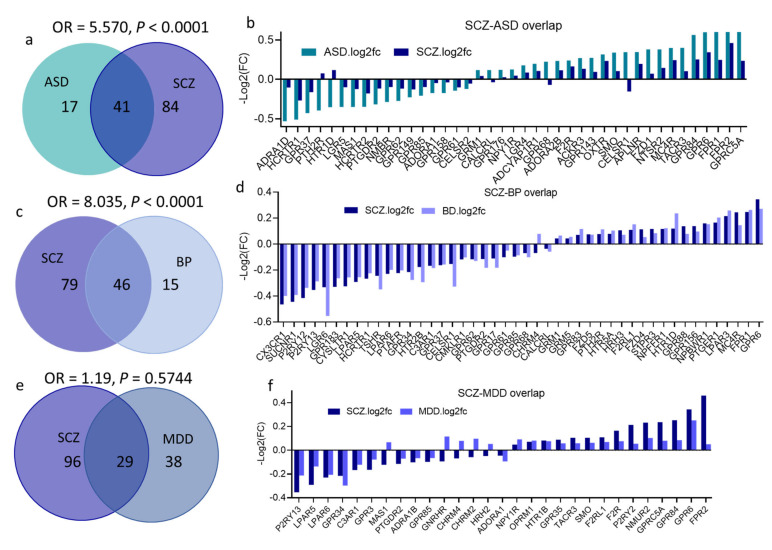

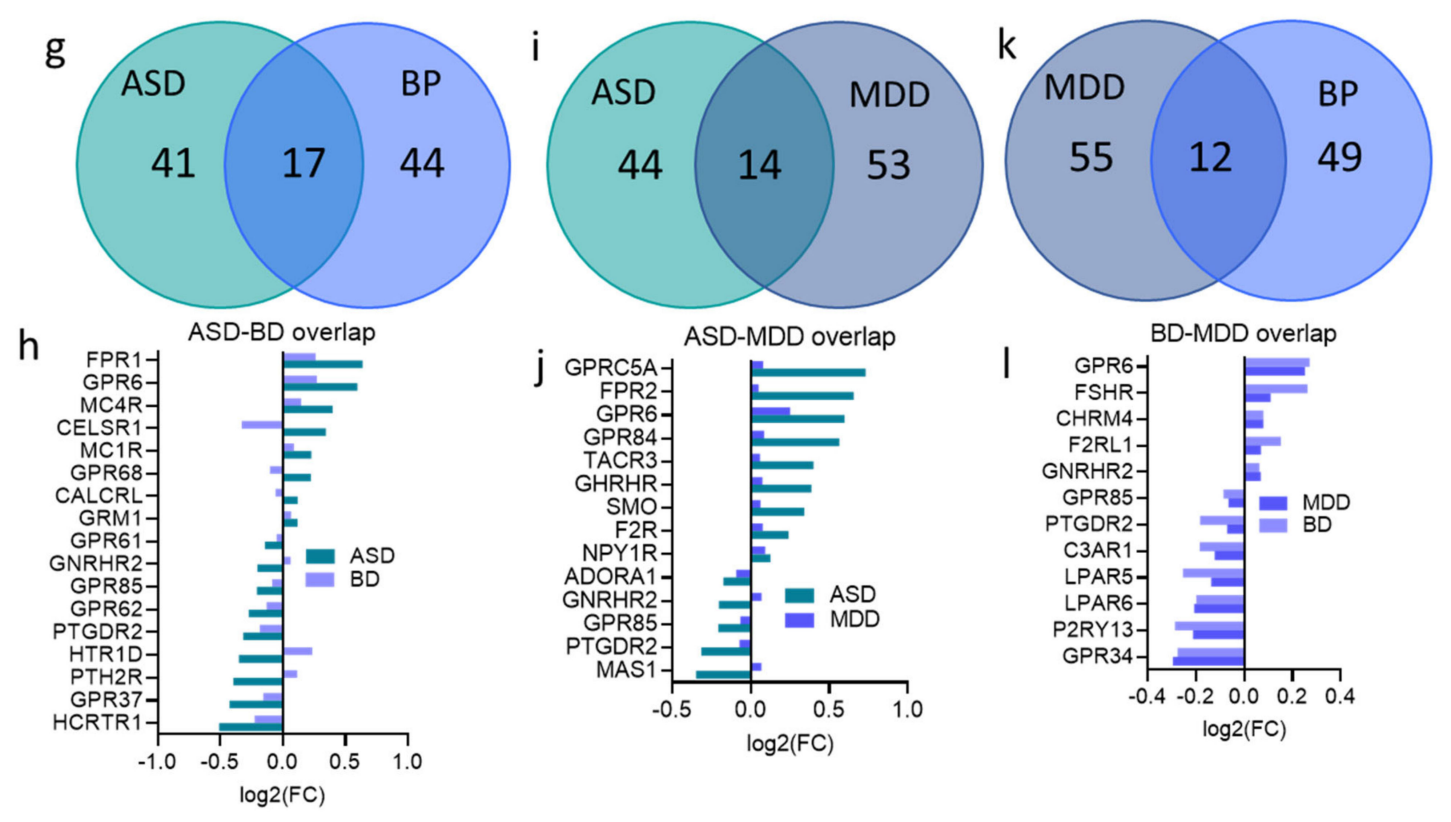
GPCRs’ DEGs overlap across the four psychiatric disorders; (**a**,**c**,**e**,**g**,**i**,**k**) Venn diagram conveying overlap between significant DEGs (*P* < 0.05) in (**a**) SCZ and ASD, (**c**) SCZ and BP, (**e**) SCZ and MDD, (**g**) ASD and BP, (**i**) ASD and MDD, (**k**) MDD and BP. Fisher’s test results are shown (OR and *P* values for each overlap). (**b**,**d**,**f**,**h**,**j**,**l**) GPCR DEGs overlapping between (**b**) SCZ and ASD, (**d**) SCZ and BP, (**f**) SCZ and MDD, (**h**) ASD and BP, (**j**) ASD and MDD, (**l**) MDD and BP.

Several GPCR DEs overlapped across the four disorders, and SCZ exhibited the highest overlap of its GPCR DEs (82 of its total 125 DEs) with at least one of the three other disorders. MDD showed the highest distinctive disease-specific GPCR gene signature, as 52% of GPCRs in MDD were uniquely dysregulated in this disorder compared to 19% and 25% of distinctive GPCR DEs in BP and ASD, respectively (Figure 5a–n).

Fisher’s exact test revealed a substantial overlap between SCZ and ASD DEs (OR = 5.26, *P* < 0.0001); approximately 70% of DEs in ASD (41 genes) were also differentially expressed in SCZ (Figure 5a). Remarkably, almost all GPCR DEs overlapped between SCZ and ASD showed a more remarkable change in ASD than in SCZ (higher log(FC), Figure 5b).

There was also significant overlap between SCZ and BP DEs (OR = 8.034, *P* < 0.0001), with 75% (46 gene) of BP DEs were also differentially expressed in SCZ (Figure 5c,d). Despite the several shared DEs among other psychiatric disorders, Fisher’s exact test revealed no significant DEs overlap between any other pairs of disorders: ASD and BP (OR = 1.4, *P* = 0.38), ASD ∩ MDD (OR = 1, *P* > 0.99), SCZ ∩ MDD (OR = 1.19, *P* = 0.57), BP ∩ MDD (OR = 0.73, *P* = 0.40) (Figure 5e–l).

We identified the common classical signaling systems mediated by the dysregulated GPCRs and found that the four disorders’ glutamate and serotonin signaling systems were disrupted (Table S2). In addition, noradrenaline signaling was disrupted in ASD, SCZ, and MDD, whereas acetylcholine signaling was disrupted in ASD, SCZ, and BP (Table S2).

Other common signaling systems mediated by dysregulated GPCRs across the four disorders included GNRH, prostaglandins, melanocortin, orexin, formyl peptide, and Wnt signaling systems disrupted in the four disorders. Photo-rhodopsin receptors were dysregulated in the four disorders, albeit distinctive rhodopsin was dysregulated across the four disorders. Parathyroid hormone and sphingosine-1-phosphate receptors were dysregulated in ASD, SCZ, and BP. Lysophosphatidic acid bound to dysregulated receptors in SCZ, BP, and MDD, whereas CGRP receptors MAS1 were dysregulated in ASD, SCZ, and BP. Neuropeptide Y receptors were dysregulated in ASD, SCZ, and MDD, and cysteinyl leukotriene receptors in SCZ, BP, and MDD (Table S2).

### 3.7. Age-Association of the GPCR DEs in the Four Psychiatric Disorders

**Figure 6:**
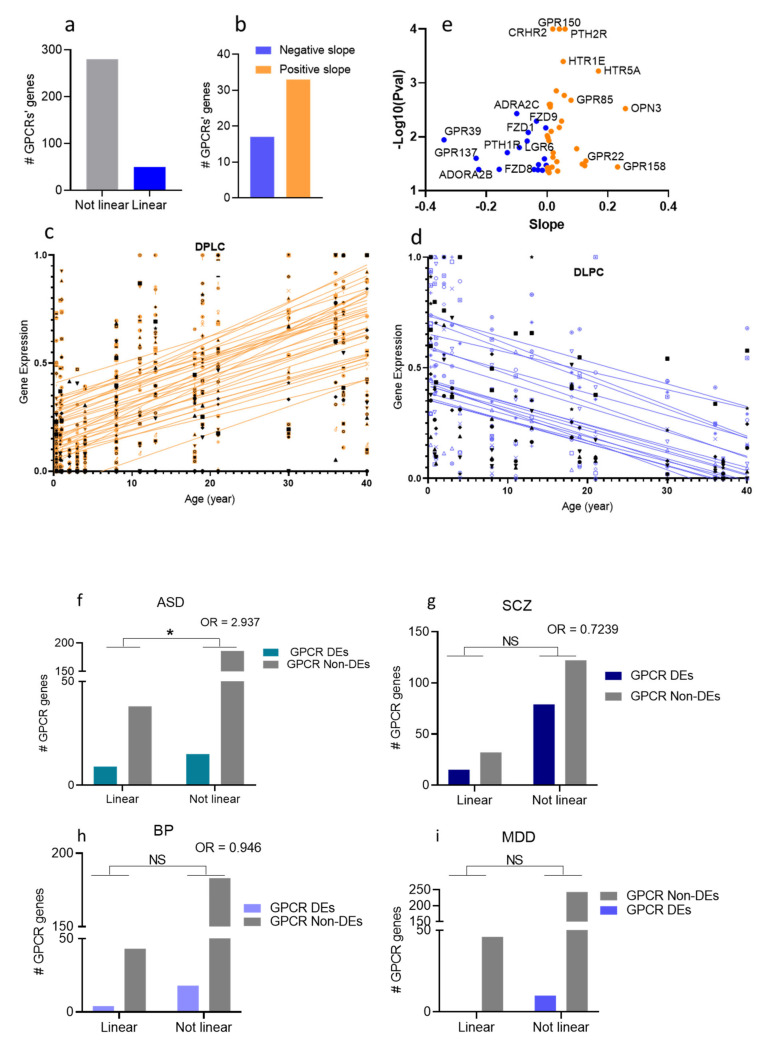
Age-dependent gene expressions of GPCRs in the human prefrontal cortex (DLPC). (**a**) Bar chart showing the numbers of GPCR genes that follow linear regression in the DLPC (**b**) Bar chart showing the numbers of upregulated GPCRs (positive slope) and downregulated (negative slope) with chronological age. (**c**,**d**) Scatter plots and fitted linear regression lines of GPCRs that were differentially expressed with age: upregulated (**c**) and downregulated (**d**) GPCRs with chronological age. (**e**) Volcano plot of regression coefficient and p-values in DLPC. The *x*-axis represents the age effect on GPCR expression measured by regression coefficient. The *y*-axis represents the -log10(*P*-value). The orange dots represent age-dependent upregulated GPCRs, and the blue dots represent age-dependent downregulated GPCRs. (**f**) Bar graph representation of the correlation between age-dependent expressions of GPCR and their dysregulations in (**f**) ASD, (**g**) SCZ, (**h**) bp, and (**i**) MDD (Fisher’s exact test shows the significant correlation in ASD, *P* = 0.02, * *P* < 0.05).

To determine whether genes encoding dysregulated GPCRs are expressed in age-dependent patterns, we acquired RNA-Seq data of GPCRs (330 GPCR transcripts) in the DLPC across the lifespan (4 months to 40 years old) [46]. We used linear regression to model gene expression levels as a function of age. We found that of the 330 GPCR transcripts, whose chronological expressions were determined in DLPC, 50 genes followed linear regression (regression coefficient at *P* < 0.05, Figure 6a). Thus, most age-associated GPCRs exhibited increased expression patterns with chronological age (Figure 6b,c). Since some GPCR genes were missing from either the lifespan or the disorders’ transcriptomic lists, the two lists were compiled, which resulted in two new lists with 248 GPCR genes in the DLPC for ASD, SCZ, and BP analyses, and 299 for MDD. Finally, we used Fisher’s exact test to test whether the dysregulated GPCRs in ASD, SCZ, BP, and MDD are associated with higher rates of linear expression change with chronological age. While there was no association between age-dependent expression of GPCRs and their dysregulations in each of SCZ, BP, and MDD, we found a significantly higher proportion of age-associated GPCR genes in the disease-associated ones compared to the random GPCR (Figure 6f–i).

## 4. Discussion

### 4.1. GPCR Expressions Are Perturbed in Four Major Psychiatric Disorders

In this study, we present a comprehensive analysis of the transcriptomic signatures of G-protein-linked signaling across four major psychiatric disorders. The results reveal that the majority (three quarters) of GPCR genes were dysregulated in ASD, SCZ, BP, and MDD. In addition, when compared with non-GPCR genes, the GPCR superfamily as a gene cluster was overrepresented in the four disorders. The analysis also identifies more extensive GPCR gene expression changes (measured as the average absolute fold change value) in the four psychiatric disorders. Moreover, the results reveal that GPCRs differentially expressed with age tend to be more dysregulated in ASD than random GPCRs (i.e., ASD-dysregulated GPCRs are more likely to follow age-related linear regression than non-dysregulated GPCRs).

Not surprisingly, most GPCR DEs in the four psychiatric disorders belonged to the A-family, which comprises most GPCRs in humans. It is noteworthy that GPCR dysregulation was most remarkable in SCZ, with around half of the GPCR genes exhibiting upregulation or downregulation. However, unexpectedly, two of the only three known adhesion GPCRs were dysregulated in ASD and SCZ, and more than 50% of frizzled GPCRs were dysregulated in SCZ.

At the G protein level, we found, not surprisingly, that the majority of GPCR DEs in the four psychiatric disorders are coupled to the Gαi/o isoform, the most common Gα in biological systems. In BP, however, we found a lesser trend of dysregulated GPCRs to couple to Gαq compared with the non-dysregulated GPCRs. Similar to GPCRs, SCZ exhibited the highest number of dysregulated G-protein isoforms.

### 4.2. Disease-Specific GPCR Transcriptomic Signatures

#### 4.2.1. ASD Is Associated with Perturbed Wnt-SMO Pathways and Neuropeptide Transmission

GPCRs dysregulated in ASD included primarily frizzled GPCRs such as FZD1, which transduce SMO signals from the Wnt lipoglycoprotein growth factors [48]. The Wnt pathway plays a critical role in regulating neuronal precursor proliferation, neuronal migration, and fate specification during neurodevelopment. It is also involved in synaptic differentiation and mature synapse modulation [49]. Thus, perturbation of GPCR components of the Wnt pathway may cause synaptic and circuit dysfunctions during neurodevelopment, which are considered to be at the center of ASD etiology. Other dysregulated GPCRs include neuropeptides’ GPCRs, particularly melanocortin receptors, orexin receptors, oxytocin receptors, neurotensin receptors, somatostatin receptors, CGRP, and FPR1/2 receptors. Of these neuropeptides, oxytocin has been extensively studied in the context of ASD [50,51,52]. PTH2R, which encodes parathyroid hormone-2 receptor, a class B GPCR, was the second most significant GPCR DEs in ASD after FZD1. PTH2R binds in the brain to the neuropeptide TIP39 (Tuberoinfundibular Peptide of 39 Residues) and is primarily expressed in the hypothalamus and amygdala, brain regions involved in social behavior and fear [53,54,55]. Anxiety is a typical overlapping symptom in ASD patients [56,57], suggesting a role for the downregulated PTHR2R gene. In support of this speculation, we found that the ADRA1D gene, which encodes the Alpha-1D adrenergic receptor (α1D-AR), was the top downregulated GPCR DE in ASD. α1D-AR, primarily found in the hippocampus and frontal cortex, exhibits strong desensitization and downregulation in response to its over-activation by noradrenaline [58,59]. Given the known role of adrenergic receptors in mediating the response to stress [60,61,62], the profound downregulation of α1B-AR in ASD may reflect a chronic over-activation by noradrenaline in response to stress exposure during developmental stages.

#### 4.2.2. GPCRs Differentially Expressed with Chronological Age Are at a Higher Risk of Dysregulation in ASD than Random GPCRs 

The onset of the four disorders varies from early life for ASD to late adolescence for SCZ and early adulthood and midlife for BP and MDD. Accordingly, we speculate that GPCRs’ dysregulations in these disorders relate to the high dynamics and changeability of the GPCR transcriptome as a function of age or in response to stimuli in critical age windows. Our speculation implies that high GPCRs’ age-dependent dynamics may lower their threshold for dysregulation. Interestingly, we found that both FZD1 and PTH2R, which exhibited the most significant expression changes in ASD, were also differentially expressed with chronological age. However, their age-dependent expression patterns were in opposite directions from the patterns of their expression changes in ASD. For instance, while FZD1 expression increased in ASD, it was negatively associated with chronological age (decreased across the lifespan), whereas PTH2R expression was decreased in ASD and was positively correlated with chronological age (increased across the lifespan).

We asked whether the apparent correlation between the age-dependent expression patterns of FZD1 and PTH2R and their dysregulation in ASD is a random observation or can be generalized to other GPCR DEs in ASD or even to other psychiatric disorders. In each of the four disorders, we identified DEs and non-DEs differentially expressed with chronological age and the direction of their expression across the lifespan. Our findings support a correlation between the age-related GPCR gene expression profiles and their tendency for dysregulation in ASD, but not other disorders. Accordingly, GPCRs differentially expressed with age have a higher propensity for dysregulation in ASD than random GPCRs, and ASD-dysregulated GPCRs are more likely to follow linear regression with age than non-dysregulated GPCRs.

Based on our results, we propose a GPCR transcriptomic instability theory in ASD, wherein GPCR transcriptomic instability (malleability) at critical developmental stages increases their tendency for dysregulation in ASD. The generalizability of this hypothesis to non-GPCR gens and other mental disorders is worth further investigation.

#### 4.2.3. SCZ: A Dopamine GPCR Disorder?

SCZ had the highest number of GPCR DEs. Compared with ASD DEs, we found that SCZ DEs were more enriched with biogenic amines and lipids transmissions, whereas peptides exhibited less enrichment in SCZ GPCR DEs than in ASD. In addition, a few GPCRs revealed SCZ-specific DEs, particularly the neuropeptides’ receptors BDKRB2, MCHR1, KISS1R, and QRFPR.

Along with embryonic linked signaling, FZDs (1–10) also regulate adult neurogenesis [63,64] and have a crucial role in regulating hippocampal neurons’ differentiation and migration [65]. Thus, the dysregulation of nine of the Wnt-SMO pathways in SCZ indicates that early developmental stages and adolescence present a critical window of vulnerability to perturbation of these pathways SCZ [66,67].

Although biogenic amines neurotransmitters were more enriched in adolescence- and adulthood-onset disorders (SCZ, BP, and MDD) compared to the early life disorder ASD, SCZ exhibited the most significant number of biogenic amines’ dysregulated GPCRs.

Notably, the dopamine GPCRs were exclusively perturbed in SCZ, with three dopamine GPCRs (DRD2, DRD4, and DRD5) exhibiting differential expression in SCZ. The adrenergic, histaminergic, and serotonergic systems were also enriched in SCZ with seven serotonergic receptors and four adrenergic receptors, two cholinergic receptors, and two histaminergic receptors being DEs in this disorder. These results provide a robust mechanistic basis for the early hypothesis of SCZ, which was centered, for many years, on the hyperactivity of the dopamine system [68,69]. This hypothesis originated from the observations that antipsychotic medications antagonize dopamine receptors and that certain drugs that enhance dopamine activity, such as amphetamine, cause psychosis in healthy individuals or exacerbate schizophrenic symptoms [70]. Due to the high potency of second-generation antipsychotics for serotonergic, histaminergic, and α-adrenergic receptors, the SCZ hypothesis evolved to implicate other GPCRs [71,72,73]. However, the dopamine system remains at the center of this theory. The distinctive dysregulation of three dopamine receptors exclusively in SCZ provides a piece of mechanistic evidence that credits dopamine theory and highlights dopamine GPCRs’ roles in the neuropathology of schizophrenia. Notably, other non-biogenic amine neurotransmitters GPCRs were also disrupted in SCZ, including the glutamate transmission with four metabotropic glutamate receptors exhibiting abnormal expressions in SCZ.

#### 4.2.4. BP as a Lipid Transmission Disorder 

The landscape of BP GPCR DEs is distinguished from the three other disorders in presenting extensive perturbed lipid transmission and a low tendency of dysregulation of GPCRs coupled to Gq/G11. The disrupted lipid pathways in BP presented those of arachidonic acid derivatives (prostaglandins, leukotrienes, and oxoeicosanoid) and lysophosphatidic acid and sphingosine. Arachidonic acid (AA) is an essential constituent of the cell membrane released from membrane phospholipids through GPCR-Gq-initiated activation of phospholipase A2 [74,75,76]. AA controls cell membrane fluidity and plays a crucial role in maintaining cell integrity and the functions of specific membrane proteins involved in cellular signaling and brain synaptic plasticity. Further, the AA-derivatives prostaglandins and leukotrienes are released in the brain and function as neuromodulators via activation of GPCRs. Arachidonic acid turnover and signaling have been implicated in BP [77,78], and the therapeutic effects of mood stabilizers such as lithium and valproic acid are partially related to their effects on arachidonic acid turnover [77,79,80].

On the other hand, the pharmacological actions of BP primary treatments are known to modulate Gαq signaling cascades, namely, DAG and IP3 secondary messengers’ pathways. Therefore, the low prevalence of dysregulated GPCRs coupling to Gq/G11 is surprising. Further, the OPRL1 gene, which encodes the nociceptin/orphanin FQ (N/OFQ) receptor, is exclusively upregulated in BP. N/OFQ levels were reported to be significantly elevated in the plasma of patients with BP [81], and OFQ receptor antagonists have been proposed as a potential treatment for BP [82].

#### 4.2.5. MDD Is Associated with Upregulated GPCRs and Disrupted Biogenic Amine Transmissions 

Unlike ASD, SCZ, and BP, GPCR DEs in the MDD exhibited more upregulation than downregulation. The amine theory of MDD suggests that the depletions of amine neurotransmitters may underlie the pathophysiology of MDD, and the therapeutic actions of current antidepressants rely on increasing monoamine levels via inhibiting their metabolism or uptake. Furthermore, the depletion of monoamine transmitters is known to cause compensatory upregulation or supersensitivity of their receptors [83,84,85,86]. Therefore, our finding that most amine GPCRs were upregulated in MDD substantiates the contribution of monoamine signaling to the MDD pathophysiology and may reveal compensatory mechanisms to counterbalance the reduced levels of the monoamine transmitters [87].

One important GPCR, exclusively dysregulated in MDD (downregulated), was CRHR1. CRHR1 mediates the action of corticotropin-releasing hormone (CRH), one of the most extensively studied systems concerning its potential role in stress and depression [88,89]. Recent data on CRHR1 antagonists suggest that this receptor might be a promising target for the treatment of MDD. Compelling evidence indicates that early stress enhances adult depressive mood through perturbed GPCRs. Early life stage represents a critical window for setting up the neurocircuits that regulate emotions in later life, which are modified in response to environmental stimuli. Early life adversity reprograms structural and functional changes in these neurocircuits, enhancing the risk for MDD. According to the early stress theory of depression, CRH-CRHR1 signaling initiates the hormonal stress-response pathway involving perturbed glucocorticoid (GC) signaling pathways [90,91]. The role of early-life stress in adulthood development of depression is also mediated through other neurotransmitters GPCRs, including glutamate, GABA, endocannabinoids, and neuropeptides, several of which are dysregulated in MDD [25].

The GPCR genes that showed the highest changes in MDD, however, were GPR6 (log2FC > 0.2) and GPR34, P2RY13, and LPAR6 (log2(FC) < −0.2). While there are no studies on the role of GPR6 in MDD, the selective localization of this receptor in the straiatum [92] and the discovery of endogenous inverse agonists of GPR6 that are dopamine derivatives (*N*-arachidonoyl dopamine, *N*-docosahexaenoyl dopamine, *N*-oleoyl dopamine, and *N*-palmitoyl dopamine) [93] suggest that GPR6 might be a possible therapeutic target for MDD. Interestingly, GPR34, which is activated by lysophosphatidylserine, LPAR6 (lysophosphatidic acid receptor 6), which is also activated by purines (P2RY5), and P2RY13 (Purinergic Receptor P2Y13) are primarily expressed in the microglia and regulate the function and morphology of microglia during neuroinflammation [94,95,96,97,98,99,100]. Our data provide further evidence for the role of neuroinflammation in MDD, particularly that 14% of GPCRs dysregulated in MDD were chemokines’ receptors.

### 4.3. Dysregulated GPCRs Overlap across Psychiatric Disorders

Not only did SCZ show the largest number of dysregulated GPCR genes, but its DEs showed the highest levels of GPCR transcriptomic overlap with ASD, BP, and MDD.

Interestingly, GPCR DEs shared between ASD and SCZ exhibited more extensive fold changes in ASD than SCZ. Initially, SCZ and BP were viewed as different phases of the same disorder, with ASD manifesting itself as an earlier phase of SCZ. Our study provides further evidence for the overlapping nature of these disorders and possible common etiological components and argues for a theory that the degree of the expression changes of GPCRs, and probably other genes, may determine whether the disorder manifests itself as ASD in early childhood or SCZ later in adolescence.

The largest proportion of dysregulated GPCRs were shared between SCZ and BP, with approximately 75% of BP GPCR DEs overlapping with SCZ. This high degree of overlap is in accordance with the abundant genetic and transcriptomic overlaps and the highly shared clinical features between SCZ and BD. Among the top shared GPCRs between BP and SCZ are SUCNR1, LGR6, and CX3CR1, which mediate the succinate, Wnt, and chemokine neurotactin signaling, respectively. These three receptor pathways are essential for microglia migration to their synaptic targets, pointing at a critical role of microglia functional disruptions in the pathophysiology of both ASD and SCZ.

Unlike the high overlap observed among ASD, BD, and SCZ, MDD exhibited lesser commonalities with the three other disorders, suggesting that the etiology of MDD is less related to those of the three other disorders. The substantial overlap of GPCR DEs among SCZ, ASD, and BP and their age-related shared clinical manifestations provide a compelling theory that ASD, SCZ, and BP can be viewed as a single “spectrum disorder,” wherein clinical manifestations vary in their explicit phenotype expression across genetically predisposed individuals.

### 4.4. Dysregulated GPCRs Are Involved in the Regulation of Sleep–Wake and Feeding

An intriguing observation was that many dysregulated GPCRs and their signaling systems were involved in sleep–wake and feeding behavior. Sleep disturbances are primary comorbid conditions across the four disorders ASD, SCZ, BP, and MDD. Most ASD patients suffer from sleep disturbance [101,102], often characterized by insomnia. Similarly, SCZ and the manic phase of BP share the reduced need for sleep [103,104,105,106]. In MDD and during the depressive phase of BP, patients present both hypersomnia and severe insomnia [107,108,109,110].

The sleep–wake cycle is modulated by a complex interaction between the different transmitter and peptide systems located throughout the brain. For example, neurotransmission systems in the brainstem and hypothalamus, including serotonin, norepinephrine, dopamine, acetylcholine, glutamate, and histamine, act together to promote the generation and maintenance of wakefulness (for review, [111,112]. On the other hand, GABA and adenosine are crucial transmitters for sleep promotion.

Neuropeptides involved in sleep–wake regulation are synthesized and released from the hypothalamic nuclei, including orexin, galanin, melanocortin, neuropeptide Y, and melanin-concentrating hormone corticotropin-releasing hormone (CRH), and somatostatin (for review, [113]). Sleep-wake cycles are highly regulated processes under the tight control of the circadian rhythm, which is synchronized to the 24-h light–dark cycle. Thus, light is the most persistent and powerful circadian entrainers across all ages. Interestingly, light/photon GPCRs (opsins) were among the dysregulated GPCRs across the four psychiatric disorders. Whether light can activate brain opsins and the functional significance of such activation are not known; however, the dysregulations of the brain light receptors, in accordance with the dysregulation of transmitters’ GPCRs involved in sleep–wake regulation, provide compelling evidence for the link between GPCRs’ dysregulation and sleep disturbances in the psychiatric disorders.

Strikingly, almost all the dysregulated GPCRs that mediate the signaling of brain transmitter systems in sleep are also known for their role in feeding behavior and body weight. Examples illustrating this view are GPCRs for the neurotransmitters noradrenaline, serotonin, and dopamine. Orexin, ghrelin, neuropeptide-Y, melanin-concentrating hormone, GHRH, endorphins, cannabinoid, galanin, and nociceptin (OFQ) are orexigenic peptides, whereas melanocortin, GLP-1, corticotropic-releasing hormone, oxytocin, neuromedin U, and neurotensin act as anorexic peptides (for review, [114,115]). Appetite changes, bodyweight loss, and obesity have frequently been associated with psychiatric disorders. In addition, there is considerable evidence for comorbidities in psychiatric disorders with eating and appetite disorders. For example, there is a profound shift in the appetitive characteristics in depression from weight loss in the depression early stage to weight gain at later stages [116,117]. Further, eating behavior disorders such as binge eating, anorexia nervosa, bulimia nervosa, and night eating have been considered characteristic features of schizophrenia (for review, [118]). Similarly, several studies have illustrated the prevalence of certain eating disorders such as anorexia nervosa and bulimia nervosa in ASD, and low body weight has often been seen in male subjects with ASD (for review, [119]). Rapid weight fluctuation is a common feature in BP; more than 5% loss or gain of body weight can be observed in one month, and overweight and disordered eating behaviors differentially impact BD patients [120,121]. Since sleep and feeding behaviors are regulated by internal circadian rhythm, our results raise an intriguing hypothesis that perturbed brain GPCR signaling may play a key role in mechanistically mediating the consequences of circadian rhythm disruption in psychiatric disorders.

In conclusion, the results of our study support the critical role of perturbed brain GPCR signaling pathways in the etiological and pathophysiological mechanisms of psychiatric disorders. Furthermore, our study suggests that common GPCRs’ signaling may mediate distinct phenotypic presentations across psychiatric disorders. Consequently, targeting these GPCRs could serve as a common therapeutic strategy to treat specific clinical symptoms across psychiatric disorders.

## Figures and Tables

**Table 1:** GPCRs’ lists included in the study. List of GPCR families and number of GPCRs included in the study from the different datasets.

Classes	# from iuphar-db.org (Accessed on 4 May 2021)	# GPCRs in SCZ, ASD, BP Transcriptomics	# GPCRs in MDD Transcriptomics
Class A	290	207	253
Class B	15	12	14
Class C	22	17	20
Frizzled	11	11	11
Adhesion	33	3	3
Others	5	5	5
Total	376	255	306

## Data Availability

Not applicable.

## Supplementary Materials

The following are available online at www.mdpi.com/article/10.3390/cells10112967/s1, Table S1. Lists of GPCRs included in the study and their classifications (References [27,47]); Table S2. List of ligated GPCRs dysregulated in the four psychiatric disorders with their ligand.
