# Supplementary material for: Transcriptome Profiling of Dysregulated GPCRs Reveals Overlapping Patterns across Psychiatric Disorders and Age-Disease Interactions
Source: Cells. 2021 Oct 31;10(11):2967. doi: 10.3390/cells10112967 (PMC8616384; doi:10.3390/cells10112967)
Supplement: Supplementary file 1 [file cells-10-02967-s001.zip › cells-1424022-supplementary/Table S2.pdf]

Ligand		
**Biogenic Amines**	**Disorder**	**Receptors**
Serotonin (5-HT)	ASD<br>SCZ<br>BP<br>MDD	HTR1D<br>HTR1B HTR1D HTR1E HTR1F HTR2B HTR5A HTR6<br>HTR2B HTR7 HTR5A HTR1D<br>HTR1B
Noradrenaline (NA)	ASD<br>SCZ<br>MDD	ADRA1D<br>ADRA1B ADRA1D ADRA2A ADRA2B<br>ADRA1B ADRB3
Acetylcholine (Ach)	ASD<br>SCZ<br>BP	CHRM5<br>CHRM2 CHRM4<br>CHRM3
Histamine (His)	SCZ<br>BP<br>MDD	HRH2 HRH3<br>HRH3<br>HRH1 HRH2 HRH4
Dopamine (DA)	SCZ	DRD2 DRD4 DRD5
Trace amines (TA)	MDD	TAAR5 TAAR8
**Amino Acids/Dicarboxylic acids**	**Disorder**	**Receptors**
Glutamate	ASD<br>SCZ<br>BP<br>MDD	GRM1 GRM8<br>GRM1 GRM2 GRM3 GRM5<br>GRM1 GRM5<br>GRM6 GRM7
Succinate	SCZ<br>BP	SUCNR1<br>SUCNR1
GABA	MDD	GABBR1
**Hormones**	**Disorder**	**Receptors**
Gonadotropin Releasing Hormone (GNRH)	ASD<br>SCZ<br>BP<br>MDD	GNRHR2<br>GNRHR<br>GNRHR2<br>GNRHR GNRHR2
Parathyroid hormone	ASD<br>SCZ<br>BP	PTH2R<br>PTH2R<br>PTH2R
Thyroid stimulating hormone (TSH)	SCZ<br>BP	TSHR<br>TSHR
Follicle Stimulating Hormone (FSH)	BP<br>MDD	FSHR<br>FSHR
Growth hormone releasing hormone (GHRH)	ASD<br>MDD	GHRHR<br>GHRHR
Prolactin Releasing Hormone	SCZ	PRLHR
Ghrelin	MDD	GHSR
**Lipids**	**Disorder**	**Receptors**
Prostaglandin	ASD<br>SCZ<br>BP<br>MDD	PTGDR2 PTGER3<br>PTGDR2 PTGDR PTGER1 PTGIR<br>PTGDR2 PTGER1 PTGER4<br>PTGDR2
Sphingosine-1-Phosphate	ASD<br>SCZ<br>BP	S1PR1<br>S1PR3<br>S1PR3
Cysteinyl Leukotriene	SCZ<br>BP<br>MDD	CYSLTR1<br>CYSLTR1<br>CYSLTR2
lysophosphatidic acid	SCZ<br>BP	LPAR1 LPAR3 LPAR5 LPAR6<br>LPAR3 LTB4R LPAR5 LPAR6

	MDD	LPAR2 LPAR5 LPAR6
Oxoeicosanoid	BP	OXER1
cannabinoids	MDD	CNR2
**Peptides**	**Disorder**	**Receptors**
Melanocortin	ASD<br>SCZ<br>BP<br>MDD	MC1R<br>MC4R<br>MC1R MC4R<br>MC2R MC5R
Orexin	ASD<br>SCZ<br>BP<br>MDD	HCRTR1 HCRTR2<br>HCRTR1 HCRTR2<br>HCRTR1<br>HCAR2
Formyl Peptide	ASD<br>SCZ<br>BP<br>MDD	FPR1 FPR2<br>FPR1 FPR2<br>FPR1<br>FPR2
Calcitonin gene-related peptide (CGRP)	ASD<br>SCZ<br>BP	CALCRL<br>CALCRL<br>CALCRL
Angiotensin-(1-7)	ASD<br>SCZ<br>MDD	MAS1<br>MAS1<br>MAS1
Neuropeptide Y	ASD<br>SCZ<br>MDD	NPY1R<br>NPY1R<br>NPY1R NPY6R
Neuromedin B	ASD<br>SCZ	NMBR<br>NMBR
Oxytocin	ASD<br>SCZ	OXTR<br>OXTR
Neurotensin	ASD<br>SCZ	NTSR1 NTSR2<br>NTSR2
Somatostatin	ASD<br>SCZ	SSTR1<br>SSTR2 SSTR4
Apelin	ASD<br>SCZ	APLNR<br>APLNR
Adenylate cyclase activating polypeptide (ADCYAP)	ASD<br>SCZ	ADCYAP1R1<br>ADCYAP1R1
platelet Activating Factor	SCZ<br>BP	PTAFR<br>PTAFR
Neuropeptide FF	SCZ<br>BP	NPFFR1<br>NPFFR1
Neuropeptides B And W	SCZ<br>BP	NPBWR1 NPBWR2<br>NPBWR1
Galanin	SCZ<br>MDD	GALR1<br>GALR3
Neuromedin U	SCZ<br>MDD	NMUR2<br>NMUR2
Relaxin	SCZ<br>MDD	RXFP1<br>RXFP3
Endorphin	SCZ<br>MDD	OPRK1 OPRM1<br>OPRM1
Vasopressin	BP<br>MDD	AVPR2<br>AVPR1B
Glucagon Like Peptide	ASD	GLP2R
tachykinin	ASD	TACR3

	SCZ<br>MDD	TACR3<br>TACR3
Endothelin	ASD	EDNRB
Bradykinin	SCZ	BDKRB2
Melanin concentrating hormone (MCH)	SCZ	MCHR1
Vasoactive intestinal peptide (VIP)	SCZ	VIPR1
Kisspeptin	SCZ	KISS1R
Pyroglutamylated RFamide Peptide	SCZ	QRFPR
Nociceptin/Orphanin QR	BP	OPRL1
Gastrin releasing peptide (GRH)	MDD	GRPR
Bile acid	MDD	GPBAR1
Corticotropin releasing hormone (CRH)	MDD	CRHR1
**Protein**	**Disorder**	**Receptors**
Wnt	ASD<br>SCZ<br>BP<br>MDD	FZD1 LGR5 LGR4 SMO<br>FZD1 FZD4 FZD5 FZD7 FZD9 LGR4 LGR5 LGR6 SMO<br>LGR6 FZD4 FZD5<br>SMO
Proteases	ASD<br>SCZ<br>MDD	F2R<br>F2R<br>F2R
Proteases	SCZ<br>BP<br>MDD	F2RL1<br>F2RL1<br>F2RL1
**Purine**	**Names**	**Receptors**
Adenosine	ASD<br>SCZ<br>MDD	ADORA1 ADORA2B<br>ADORA1 ADORA2B<br>ADORA1
ATP/ADP	SCZ<br>BP<br>MDD	P2RY12 P2RY13 P2RY2<br>P2RY12 P2RY13<br>P2RY13 P2RY2
**Photon**	**Disorder**	**Receptors**
Photon	ASD<br>SCZ<br>BP<br>MDD	OPN3<br>RHO<br>OPN4<br>OPN5
**Adhesion**	**Disorder**	**Receptors**
Unknown	ASD<br>SCZ<br>BP	CELSR2 CELSR1<br>CELSR1 CELSR2<br>CELSR1
